# Prevalence of Orthostatic Hypertension in Elderly Patients with Type 2 Diabetes

**DOI:** 10.1155/2015/463487

**Published:** 2015-05-20

**Authors:** Patrícia Mesquita, Deborah Queiroz, Vanderson Lamartine de Lima Silva, Vanessa de Carvalho Texeira, Yasmin Rodrigues Vilaça de Lima, Edinaldo Rodrigues Fontes Júnior, Jéssica Garcia, Francisco Bandeira

**Affiliations:** Division of Endocrinology, Diabetes and Bone Diseases, Agamenon Magalhaes Hospital, University of Pernambuco Medical School, Rua Raimundo Freixeiras 47, 52070-020 Recife, PE, Brazil

## Abstract

*Background*. The aim of the present study was to determine the prevalence of orthostatic hypertension (OHT) in elderly patients with type 2 diabetes and its relation to metabolic and echocardiographic parameters. *Methods*. This was an analytical cross-sectional study in 97 patients normotensive or hypertensive. OHT was defined as a ≥10 mmHg increase in systolic blood pressure after four minutes in the standing position. *Results*. The prevalence of OHT was 20.6%. The mean body mass index was significantly higher in patients with OHT than in those without it (29.80 ± 4.10 versus 27.51 ± 3.98 kg/m^2^; *P* = 0.026). There were no statistically significant differences between the two groups for other metabolic parameters. Among the 68 patients who had an echocardiographic examination 27% of those with OHT had an increase in their left atrial volume index (LAVi) compared with 75% of those who did not have OHT (*P* = 0.004). The mean LAVi of patients with OHT was significantly lower than that of those without OHT (26.27 ± 6.37 versus 32.65 ± 7.54, resp.; *P* = 0.011). *Conclusion*. We found a high prevalence of orthostatic hypertension and a lower left atrial volume indexed in the patients with orthostatic hypertension.

## 1. Introduction

Autonomic cardiac neuropathy is associated with significant morbidity and mortality in diabetics, the risk being greater in those with hypertension [1]. Some reports in the literature suggest that orthostatic hypertension occurs in some forms of autonomic dysfunction and in primary chronic conditions such as type 2 diabetes mellitus, elderly hypertensive individuals, and peripheral neuropathy [2].

The prevalence and clinical significance of orthostatic hypertension in diabetic patients are as yet not well understood, unlike postural hypotension, which is a common feature of advanced autonomic neuropathy in type 2 diabetes mellitus (T2DM) [3, 4].

The difficulty in determining the actual prevalence of orthostatic hypertension is due to several factors, such as the varying definitions used and differences between the populations that have been studied to date. There are studies that define orthostatic hypertension as a rise in systolic blood pressure (SBP) of 5 mmHg [5], while others define it as an increase in SBP of 20 mmHg [6]. Still others define it as an increase >140 mmHg in systolic and/or 90 mmHg in diastolic blood pressure (DBP) after standing up [3].

In hypertensive patients, the prevalence and clinical significance of orthostatic hypertension also remain largely undetermined [6, 7]. A recent study evaluated the association of orthostatic hypertension with cardiovascular disease (CVD) and damage to the target organ in 4,711 hypertensive and 826 normotensive patients. After controlling for confounders such as age and gender OHT was independently associated with peripheral arterial disease (OR 1.36, 95% CI 1.05 to 1.81; *P* < 0.05) and stroke (OR 1.76, 95% CI 1.27 to 2.26; *P* < 0.01) [6].

In a study of 277 Japanese males with DM2, including 90 hypertensive patients and 128 nondiabetic males matched by age, excluding users of antihypertensive medications, the prevalence of orthostatic hypertension, in normotensive and hypertensive diabetics, was significantly higher than in the controls (12.8 versus 1.8%; *P* < 0.01, for normotensive patients, and 12.6 versus 11.1%, nonsignificant for hypertensive patients) [3]. A population-based study revealed a positive correlation of postural hypotension/hypertension and orthostatic dizziness in adults (≥20 yr) of all age groups, defining OHT as a postural increase in SBP ≥20 mmHg and that age (*P* < 0.001) and systolic blood pressure in the supine position (*P* = 0.023) were correlated with orthostatic hypertension in a statistically significant manner, with the risk for this condition increasing in adults from the age of 40 yr [8].

In view of the lack of published data on the prevalence of orthostatic hypertension in elderly patients with diabetes, this study was designed to determine the prevalence of orthostatic hypertension and its possible relation to metabolic factors and echocardiographic parameters in a population referred for treatment to an outpatient endocrine clinic.

## 2. Subjects and Methods

Approximately 4,800 patients spontaneously sought the outpatient endocrinology clinic at Agamenon Magalhães Hospital within six months of data collection. Assuming a 12% prevalence of orthostatic hypotension in normotensive and hypertensive diabetics, a sample of 375 patients with an error rate of 2.5% was calculated. However only 97 patients 60 years and older were evaluated, all of whom were diagnosed with type 2 diabetes mellitus. The patients were normotensive or hypertensive, receiving either angiotensin-converting enzyme inhibitors or angiotensin receptor blockers and/or a calcium channel blocker. The exclusion criteria were as follows: patients with type 1 diabetes mellitus; those reporting the chronic use of diuretics, beta blockers or alpha blockers for treatment of hypertension; heart failure patients with systolic ejection fraction ≤35%, or functional class III or IV as established by the New York Heart Association; patients with atrial fibrillation; patients suffering from terminal chronic renal disease undergoing hemodialysis; patients with cancer at any site except the skin (nonmelanoma); those with rheumatic diseases (rheumatoid arthritis or arthrosis of the hands and feet); patients with limitations in the supine position; and those with Parkinson's disease or tremors of the extremities.

The patients answered a questionnaire after signing the informed consent form. The questionnaire consisted of age, sex, physical examination with measurement of weight, height, waist circumference, and hip circumference, physical examination of the feet (tactile, thermal and vibratory sensitivity, presence or not of ulcer, and test monofilament), medications used, diagnosis of arterial hypertension, and time since diagnosis of diabetes mellitus. They were also evaluated for the presence of comorbidities, based on their previous diagnosis of cardiovascular disease due to acute myocardial infarction or coronary artery bypass graft (with bypass surgery or stenting) coronary angiography with stenosis of at least ≥50%, ischemic stroke, ≥50% stenosis of the carotid, or previous peripheral arterial disease.

Patients underwent a thorough physical examination, including measurement of systolic blood pressure (SBP) and diastolic blood pressure (DBP) with an automatic device (Omron Health Care, Inc., Kyoto, Japan), weight, height, body mass index (BMI), waist circumference, and hip circumference.

Blood pressure was measured with the patient seated and again after four minutes in the standing position, using an automatic device (Omron Health Care, Inc., Kyoto, Japan). Two measurements were taken consecutively in seated and standing with the arm supported at heart level in both positions, the mean of the two being considered for purposes of assessment.

Blood samples were collected after 12 hours of fasting to determine serum levels of glucose, glycated hemoglobin, total cholesterol and fractions (LDL-cholesterol and HDL-cholesterol), triglycerides, and creatinine.

Urine samples were collected for creatinine and protein determination.

Laboratory tests were performed using an autoanalyzer, Johnson & Johnson, USA, and for HbA1C, Fugion, USA.

Patients underwent echocardiography (Philips Envisor USA, 2005), conducted by an examiner blinded to which patients had orthostatic hypertension. Measurements were made for left atrial volume and left ventricular mass.

### 2.1. Definition of Concepts of Variables Studied

- Orthostatic hypertension was defined as a ≥10 mmHg increase in systolic blood pressure after 4 minutes in the standing position, compared with the sitting position.
- According to the recommendations of the Committee of the American Society of Echocardiography (ASE) 2005 [9] the left atrial volume index (LAVi) was measured, which is the left atrial volume divided by the body surface area. Normal volumes were considered to be from 16 to 28 mL/m^2^. LAVi greater than 28 mL/m^2^ was considered to be altered. The left ventricle mass index (LVMi) was measured, which is the mass of the left ventricle divided by the body surface area. The normal female value was from 43 to 95 g/m^2^, slightly abnormal between 96 and 108 g/m^2^, moderately abnormal between 109 and 121 g/m^2^, and critically abnormal ≥122 g/m^2^. For male it ranged from 49 to 115 g/m^2^, slightly abnormal between 116 and 131 g/m^2^, moderately abnormal between 132 and 148 g/m^2^, and critically abnormal ≥149 g/m^2^.

The study was approved by the Agamenon Magalhães Hospital Ethics in Research Committee (registration CAAE-.0114.0.236.000-11).

### 2.2. Statistical Analysis

In the statistical analysis the paired Student's *t*-test was used for numeric variables, Pearson's chi-square test for association of two categorical variables, or Fisher's exact test when it was not possible to use the chi-square test, and the Student's *t*-test demonstrated both equal and unequal variances. Verification of the hypothesis of equality of variances was performed using Levene's *F*-test and the correlation analysis using the *F*-test (ANOVA). Considering the confidence interval of 95%, the index of significance was set at *P* < 0.05. The software used was version 17 of the SPSS.

## 3. Results 

Ninety-seven patients were studied with ages ranging from 60 to 88 years (median, 68.97 yr, SD ± 6.80), of whom 30.9% (30 patients) were males. The mean time since the diagnosis of diabetes was 12.23 years (±8.34 years). Mean glycated hemoglobin was 7.9% (±1.71%). 59 (60.8%) patients were taking statins and 33 (34%) patients used insulin. Of the 97 patients, 7 (7.2%) had an ulcer on foot, 34 (24.7%) had negative monofilament test, 35 (36.1%) had a change (decreased or absent) in vibration sensitivity, 39 (40.2%) had changes (decreased or absent) in thermal sensitivity, and 11 had changes (decreased or absent) of tactile sensitivity. The majority of patients in this study had hypertension (77; 79.4%), were overweight or obese (77; 79.3%), with a mean BMI of 27.95 kg/m^2^ (Table 1). The prevalence of orthostatic hypertension was 20.6% (20 patients) (Table 1) with most patients being overweight or obese (BMI ≥ 25 kg/m^2^) (19 patients, 52.5%; *P* = 0.111) (Table 2). BMI was significantly higher in individuals with OHT than in those without (29.80 ± 4.10 versus 27.51 ± 3.98 kg/m^2^, *P* = 0.026). No statistically significant association was seen between orthostatic hypertension and abnormal physical examination of the feet. Of the orthostatic hypertension patients, 17 (22.7%) had a diagnosis of systemic arterial hypertension (*P* = 0.551) (Table 2). Of the 68 patients who did an echocardiographic examination, bearing in mind that 29 patients did not have the examination, 27% (3 patients) from those with OHT had an increase in their left atrial volume index (iLAV) compared with 75% (43 patients) of those without OHT (*P* = 0.004). The mean iLAV of patients with OHT was significantly lower than those without it (26.27 ± 6.37 versus 32.65 ± 7.54, resp., *P* = 0.011). We examined the interaction of BMI with the iLAV and found that the correlation persisted (−0.092; *P* = 0.452) (Table 3).

## 4. Discussion

Available data shows a strong association between obesity and hypertension. In the Framingham study, the relative risk of hypertension in overweight men and women was 1.46 and 1.75, respectively, after adjusting for age [10].

Data on OHT are not well characterized in the literature and its real clinical significance is unknown, although some studies carried out in recent years have suggested that orthostatic hypertension may be a new cardiovascular risk factor [11].

Orthostatic hypertension can vary from a simple incidental finding during a physical examination to a significant increase in blood pressure, resulting in baroreflex deregulation [2]. However its precise mechanism remains unclear [11].

Initial studies involving orthostatic hypertension showed that individuals with this condition had a greater decrease in cardiac output in the upright position, with an increase in venous blood flow in the lower extremities, and higher levels of plasma norepinephrine after standing up [12, 13]. The hypothesis was that the accumulation of venous blood leads to a reduction in cardiac output and that the response would be an increase in sympathetic activity, leading to elevated DBP. This hypothesis of venous pooling in the feet and subsequent decreased cardiac output initially seems paradoxical. Maybe in patients with orthostatic hypertension the central sympathetic excitation is pathologically excessive. This process could take place in an environment of partial dysautonomia involving venous capacitance or in individuals with pathological deregulation in the brainstem or centers involved in autonomic control. Why some patients experience orthostatic hypotension while others demonstrate orthostatic hypertension in this situation remains unclear [2].

In the present study orthostatic hypertension was found to be present in 20.6% of the elderly diabetic patients. In the literature the prevalence of orthostatic hypertension varies greatly, depending on the definition used and the population studied. In a study of Japanese men with diabetes, the prevalence of orthostatic hypertension was 12.8% in normotensive patients and 12.6% in hypertensives ones, using an increase in SBP of the 20 mmHg or more after 3 minutes in the supine position to define orthostatic hypertension [6]. In another study in young adults, orthostatic hypertension was defined as an increase of 5 mmHg in SBP upon standing, and the prevalence of orthostatic hypertension was 16.2% [5]. Regarding type 2 diabetic patients, the prevalence of orthostatic hypertension, defined as blood pressure (BP) <140/90 mmHg in the supine position, and BP measured again after 1 minute standing of 140/90 mmHg or more, the prevalence of HTO was 12.6% [3].

In our study, with a population consisting of patients aged 60 years and more, we found no association between orthostatic hypertension and increase in age. However, there are some reports in the literature in which older people have a higher occurrence of orthostatic hypertension [8]. Likewise, others have found that a complication of orthostatic hypertension in diabetic patients may be associated with increased serum triglyceride concentrations [2]. Similar findings were not shown to be statistically significant in this study.

In a multivariate analysis, high BMI and not being treated with insulin were independent factor associated with OHT.

The literature consulted indicates an increased occurrence of orthostatic hypertension in elderly patients with hypertension [14]. However, in the present study on diabetic patients, no significant association was observed between orthostatic hypertension and high blood pressure.

Increased left atrial volume is often reported with age and with a variety of cardiovascular disorders [15], as in hypertensive patients [16]. In our study the occurrence of orthostatic hypertension and increases in left atrial volume index were not substantiated. Our finding of a lower iLAV in patients with OHT is due to unknown mechanism. One possibility is that our patients with OHT had a significant high BMI but had a lower iLAV, suggesting that OHT may not be a harmful phenomenon. They should therefore not be submitted to an intensive control of blood pressure, considering that the results of the ACCORD BP trial in diabetics [17] fail to show any reduction in the rate of major fatal or nonfatal cardiovascular events, even with an intensive control of their blood pressure.

A study on orthostatic changes in blood pressure and target organ damage showed no significant correlation between left ventricle mass index and the occurrence of orthostatic hypertension [14], no such association been observed, as in the present study.

Our study had three main limitations: the small size of the sample; the fact that 29 patients did not undergo echocardiography exam because it had to be done on a different day from the clinical examination of the patient; and the lack of a control group.

## 5. Conclusion

We found a high prevalence of orthostatic hypertension in elderly patients with type 2 diabetes. High BMI and not being treated with insulin were independent factors associated with OHT. A left atrial volume indexed was found significantly lower suggesting that OHT may not be a harmful phenomenon.

## Figures and Tables

**Table 1:** Clinical, laboratorial, and echocardiography characteristics of studied subjects.

Variable	Mean
Age (years)	68.97 ± 6.80
Gender	
Male	30.9%
Female	69.1%
Diabetes duration (years)	12.23 ± 8.34
BMI (kg/m^2^)	27.95 ± 4.06
Waist circumference (cm)	98.45 ± 12.43
Hip circumference (cm)	101.71 ± 9.85
Fasting glucose (mg/dL)	143.20 ± 56.41
HbA1C (%)	7.90 ± 1.71
MDRD (mL/min/1.73 m^2^)	91.85 ± 25.98
HDL (mg/dL)	52.91 ± 22.73
LDL (mg/dL)	97.95 ± 31.95
Triglycerides (mg/dL)	136.61 ± 58.75

Medications	%

Statin	60.8
ACE inhibitor	49.5
ARB	23.7
CCB	19.6
Aspirin	52.6
Metformin	76.3
Sulfonylurea	32
Insulin	34

Comorbidities	

Hypertension	79.4
Stroke	4.1
Myocardial infarction	3.1
CABG	2.1
PVD	1
Left atrial volume index (mL/m^2^)	121.77 ± 22.81
Left ventricular mass index (g/m^2^)	31.60 ± 7.64
Orthostatic hypotension (DSBP > 20 and/or DDBP > 10 mmHg)	3.1%
Orthostatic hypertension (DSBP ≥ 10 mmHg)	20.6%

ACE: angiotensin converting enzyme.

ARB: angiotensin II receptor blockers.

CCB: calcium channel blockers.

CABG: coronary artery bypass grafting.

PVD: peripheral vascular disease.

BMI: body mass index.

DSBP: differential systolic blood pressure.

DDBP: Differential diastolic blood pressure.

**Table 2:** Evaluation of the occurrence of orthostatic hypertension (OHT) according to clinical data.

Variable	Occurrence of OTH		Total		*P* value	RP (CI of 95%)
	*N*	%	*N*	%		
Diabetes duration (years)						
<10	9	27.3	33	100.0	*P* ^(1)^ = 0.677	1.25 (0.48 to 3.26)
10 to 19	5	17.9	28	100.0		0.82 (0.27 to 2.49)
>19	5	21.7	23	100.0		1.00
**Group total**	**19**	**22.6**	**84**	**100.0**		
BMI						
Overweight	8	29.6	27	100.0	*P* ^(1)^ = 0.111	∗∗
Obese	11	22.9	48	100.0		∗∗
Normal	1	5.0	20	100.0		∗∗
HBP						
Yes	17	22.7	75	100.0	*P* ^(2)^ = 0.551	1.51 (0.49 to 4.65)
No	3	15.0	20	100.0		1.00
Left atrial volume index (mL/m^2^)						
Abnormal	3	6.5	46	100.0	*P* ^(1)^ = 0.004^*^	1.00
Normal	8	36.4	22	100.0		5.58 (1.64 to 19.00)
Left ventricular mass index (g/m^2^)						
Severe	4	16.0	25	100.0	*P* ^(1)^ = 0.408	∗∗
Moderate	3	20.0	15	100.0		∗∗
Slightly above normal	—	—	11	100.0		∗∗
Normal	4	23.5	17	100.0		∗∗
**Group total**	**11**	**16.2**	**68**	**100.0**		

∗Significant association at the 5.0% level.

∗∗Impossible to determine due to the occurrence of null and very low incidence rates.

(1) Using Fisher's exact test.

(2) Using Pearson's chi-square test.

BMI: body mass index.

HBP: high blood pressure.

**Table 3:** Statistics on left atrium volume according to BMI classification.

Statistics	BMI classification			*P* value
	Normal	Overweight	Obese	
(i) Mean	34.84	30.06	31.62	*P* ^(1)^ = 0.118
(ii) Median	32.74	30.17	30.76	
(iii) Standard deviation	8.85	6.01	8.62	
(iv) Minimum value	21.35	17.93	19.62	
(v) Maximum value	55.60	39.47	60.08	

(1): *F*-test (ANOVA).
